# Children admitted to hospital following unintentional injury: perspectives of health service providers in Aotearoa/New Zealand

**DOI:** 10.1186/1472-6963-10-333

**Published:** 2010-12-07

**Authors:** Shanthi Ameratunga, Sally Abel, Sandar Tin Tin, Lanuola Asiasiga, Sharon Milne, Sue Crengle

**Affiliations:** 1 Section of Epidemiology & Biostatistics, School of Population Health, Faculty of Medical & Health Sciences, University of Auckland, Auckland, New Zealand; 2 Te Kupenga Hauora Māori (Section of Māori Health), School of Population Health, Faculty of Medical & Health Sciences, University of Auckland, Auckland, New Zealand; 3 The Centre for Social and Health Outcomes Research & Evaluation, Massey University, Auckland, New Zealand

## Abstract

**Background:**

Unintentional injuries are the leading cause of death and hospitalisation among New Zealand children, with indigenous Māori and ethnic minority Pacific children significantly over represented in these statistics. International research has shown that many children hospitalised for injury, as well as their families experience high levels of stress, and ethnic disparities in the quality of trauma care are not uncommon. The research on which this paper is based sought to identify key issues and concerns for New Zealand's multi-ethnic community following hospitalisation for childhood injury in order to inform efforts to improve the quality of trauma services. This paper reports on service providers' perspectives complementing previously published research on the experiences of families of injured children.

**Methods:**

A qualitative research design involving eleven in-depth individual interviews and three focus groups was used to elicit the views of 21 purposefully selected service provider key informants from a range of professional backgrounds involved in the care and support of injured children and their families in Auckland, New Zealand. Interviews were transcribed and data were analysed using thematic analysis.

**Results:**

Key issues identified by service providers included limited ability to meet the needs of children with mild injuries, particularly their emotional needs; lack of psychological support for families; some issues related to Māori and Pacific family support services; lack of accessible and comprehensive information for children and families; poor staff continuity and coordination; and poor coordination of hospital and community services, including inadequacies in follow-up plans. There was considerable agreement between these issues and those identified by the participant families.

**Conclusions:**

The identified issues and barriers indicate the need for interventions for service improvement at systemic, provider and patient levels. Of particular relevance are strategies that enable families to have better access to information, including culturally appropriate oral and written sources; improve communication amongst staff and between staff and families; and carefully developed discharge plans that provide care continuity across boundaries between hospital and community settings. Māori and Pacific family support services are important and need better resourcing and support from an organisational culture responsive to the needs of these populations.

## Background

Unintentional (accidental) injuries are the leading cause of death for New Zealand children aged one to fourteen years, accounting for 35% to 43% of deaths in this age group [1]. The associated annual mortality rate is among the highest in high-income countries [2]. Serious non-fatal injuries account for almost 14,000 hospitalisations each year [1] imposing a substantial burden on the children involved and their families. Overseas research indicates that many children whose injuries are severe enough to warrant hospitalisation experience long-term impairments in physical and cognitive function, behavioural difficulties, and psychological distress [3-9]. The psychological impact on parents and siblings can also be considerable [7-11] with many caregivers experiencing employment and financial hardships [12,13].

The period of acute care of seriously injured children in hospital is of particular significance in this context. High quality hospital care is estimated to reduce child injury mortality rates by eight percent [14]. This phase also provides families the opportunity to gain valuable knowledge regarding the treatment their children required and services and support systems that can enable optimal recovery following discharge from hospital. Unmet needs in these areas are therefore important concerns relating to the quality of trauma care.

A study from the United States found that parents of hospitalised injured children experienced problems regarding the content and timing of communication during acute care of their children and difficulties in accessing and understanding the system of health care [15]. These concerns can be compounded by racial and ethnic disparities in the quality of trauma care that can occur in the context of broader social and economic inequities [16]. This is particularly important in New Zealand where significant socioeconomic and ethnic disparities exist in the burden of childhood injury with disproportionately higher representation of Māori and Pacific children among injury hospitalisations [17-19]. Māori (the indigenous people) comprise 15% and Pacific peoples (a range of groups who have migrated over decades from the Pacific region) 7% of the over four million people residing in New Zealand in 2006 [20] and both groups experience significantly poorer outcomes in most health, social and economic indices [21]. Although New Zealand studies have explored the concepts and perceptions relating to unintentional injuries amongst Māori and Samoan families [22,23], there is a gap in research knowledge regarding the perspectives of those who provide health and support services to injured children.

In order to address this gap, the present study explored the perspectives of 21 service providers from a range of professional backgrounds regarding the key issues facing injured children and their families accessing their services as well as other services with which the families interacted. This research was part of a larger study investigating the health and social impact of childhood injuries, and complements an exploration of the perspectives of families whose children were admitted to hospital, reported elsewhere [24].

## Methods

To ensure an appropriate research design and effective participant engagement, the multi-ethnic research team was guided by a steering group comprising a range of professional and cultural experts. The study was approved by the Auckland Ethics Committee, New Zealand.

### Design, setting and participants

A qualitative research design involving in-depth individual interviews or small focus groups was used as this was considered the most appropriate means of obtaining in-depth, descriptive detail about the roles, experiences and views of study key informants [25]. For the purposes of our study key informants were defined as key people from a range of professional disciplines and organisations involved in the care and support of injured children and their families in Auckland, New Zealand. They were selected purposefully using maximum variation sampling to ensure a wide range of expertise and experience was represented [26] and were identified through the networks of the study investigators and steering group members. They included representatives of emergency medical, trauma, general and specialist surgical services, Māori and Pacific Family Support services, therapy services, hospital school educators, rehabilitation service providers (public and private), the disability support sector and the Accident Compensation Corporation (ACC). Potential participants were approached in the first instance by phone, email or in person by one of the study investigators. As the Maori Family Support service, the Pacific Family Support service and the hospital school educator service each comprised a small team, all their team members were invited to participate. All those approached agreed to participate in the study.

Eleven individual interviews and three focus groups involving ten participants (in total) were conducted face to face using a semi-structured discussion guide. Individual interviews were used in situations where the participant's role was distinctive from other participants. Three distinct focus groups were used for the Māori Family Support team (Kaitiaki), the Pacific Family Support team and the hospital school educators as these participants worked in teams that had similar roles and responsibilities.

### Interview process

The interviews and focus groups were undertaken during March to May 2002 by six researchers. Nine of the eleven interviews were undertaken by the first two authors (SA, SA), the remaining two interviews and the Kaitiaki service focus group by two Māori researchers with medical backgrounds, the Pacific Family Support team focus group by the Pacific family researcher (LA), and the school focus group by the first author (SA) and a study investigator with strong school links. Interviews took place at a location chosen by the participants and in all cases this was their workplace. The purpose and process of the research were reviewed at the commencement of the interview and key informant(s) were given time to ask questions and clarify points. Written consent was then obtained. Interviews/focus groups took approximately 30-60 minutes and were audio-taped where participants gave permission. Those in one focus group did not wish to be audio-taped so comprehensive notes were taken.

The key informants' discussion guide was developed by the investigator team in conjunction with the advisory group. It comprised a small set of common questions that were then fashioned to suit the individual's or group's particular area of expertise and were broad, generic, open-ended and exploratory in nature. After establishing an understanding about the services provided by participants, these questions explored their views regarding how injured children and their families' needs were met by their services, and a deeper exploration of the key concerns they had about their services or other services with which these families interacted.

### Data Analysis

The taped interviews were transcribed and, where requested, a copy of the transcript was sent back to the participant to review and, if desired, add comment. No amendments were requested. Thematic analysis [26] was then undertaken whereby the data were analysed inductively by identification of fundamental ideas or meaning units in the transcripts which were then coded into sub-themes and further amalgamated into a number of themes by the second author (SA). A first draft of key themes was circulated amongst the five other interviewing researchers and revisions made based on feedback. A later draft was also discussed in depth with the whānau/family researchers, comparing findings with those from the whānau/family interviews, published previously [24].

## Results

In total, the views of 21 key informants were elicited, representing a range of professional groups. These were: paediatricians (3), hospital nurses (2), occupational therapists (2), a physiotherapist, a play therapist, a trauma coordinator, an Accident Compensation Corporation case worker, Maori whānau/family support workers (4), Pacific family support workers (3) and hospital and community-based teachers (3). All Maori whānau/family support workers and two other participants were Māori (n = 6) and all Pacific family support workers were of Pacific ethnicity (n = 3). All other participants were Pākehā (n = 12). Three of the participants were based in the community and all others were based either at the Starship Children's Hospital or KidzFirst Hospital, the major children's hospitals in the Auckland region.

A number of themes arose out of the key informant interviews and focus group discussions. Since only one key informant spoke about events prior to admission to hospital and did not mention any concerns, the key issues and concerns discussed in this paper are focused on the hospital and community environments. As the key informants were from a range of services and professions and they engaged with injured children and their whanau/families in different ways and at different points along the continuum of care, they each made relevant contributions in certain areas more than others. It was this breadth of key informants' experience and views that the study sought to capture. The theme descriptions incorporate the views and experiences of those participants with the relevant experience and quotes have been selected that best articulate and illustrate the theme components.

### Difficulty meeting the range of needs of injured children

A dominant theme was that the hospital system in particular and the health system in general did not adequately meet the needs of children with *mild *injuries. These children had less contact with all staff, particularly those of the allied professions, physiotherapy, occupational therapy and social workers. This was largely attributed to their relatively rapid transit and discharge from hospital and the obvious and justifiable prioritising of more serious cases in a situation of limited staffing, resources and bed space.

'The ward that they come to is a very high turn-over ward... obviously staffing levels are an issue. You are chasing your tail to look after the unstable patients, so the ones that are perhaps in for a minor type injury, these parents are there, the reality is they are not top of your list.' (Hospital nurse)

Even if a child's immediate physical injuries were reasonably well assessed and dealt with in the hospital setting, it was acknowledged that their emotional needs and concerns were usually not. Sometimes the child's physical injury might be relatively minor but if they were considered responsible for injuring others or if they had witnessed a serious motor vehicle crash, participants felt their emotional trauma could be extensive.

'They may have been in a car accident and although they [injuries] are actually of a minor nature, they are part of an event that may have resulted in death or injury. The child themselves may have caused an incident that may have severely injured another child and they themselves, their injuries were of a minor nature. So they [injuries] don't necessarily happen in isolation.... And I don't know how well they get picked up or followed up.' (Hospital nurse)

Some informants perceived the lack of paediatric-trained staff in the emergency department and the poor understanding of developmental stages, even amongst some staff who had paediatric training, important issues in a context where the needs of children would differ according to their developmental stage.

'[We need to] strengthen people's knowledge about the developmental issues that happen around children, then we provide a much more supportive environment for children ... An adult nurse has quite different skills to a paediatric nurse and many of the emergency departments around the country will have predominantly emergency nurses who are adult trained, so you look through different eyes.'(Play therapist)

Accommodating the particular needs of adolescents was also considered sometimes difficult as these varied greatly from those of younger children and even varied between early and late adolescence.

### Dealing with the psychosocial needs of families

Hospital-based informants identified influences that placed substantial emotional and psychological stresses on families when their child was hospitalised. These included: the family's previous experience of hospital, whether they were involved or had been hurt in the accident, whether they felt responsible for the accident, fear about how to cope with other children at home, fear about money or the need for time off work because of their child's injury and other issues going on in their lives. These multiple demands were perceived to place enormous pressures on individuals and relationships within families.

'I think [staff] under-estimate the impact of these sorts of things on family dynamics. Mothers have farmed off other children and they work. So for any acute hospitalisation there's quite widespread impact on the family, even if a child is only in for 24 hours. And I think sometimes we are a bit unsympathetic.'(Hospital nurse)

There was strong empathy for the onus of responsibility felt by sole parents.

'I think it's very tough on single-parent families. They have to take it all on board and make all the decisions...Having to make the decisions and being responsible for when to do things and what to do and how to plan it and organise it all with the rest of the [extended] family and things like that.' (Physiotherapist)

Practical considerations, such as the difficulty and cost of parking and access to meals, became important issues for families dealing with these stresses.

'I think dishing out the old taxi voucher to get families home or to get other family members in, all those sorts of things that are quite small, really help... And food. Kids coming in acutely... their mothers haven't had anything to eat and there's no food for them and if they haven't brought money with them... All those sorts of things add stress for the family'. (Hospital nurse)

Services were perceived to deal very poorly with the emotional and psychological impact of trauma on the families. Key informants acknowledged that many parents experienced considerable guilt and anger because they had not prevented their child's injury or were afraid that it might appear that they were careless or abusive. Several informants mentioned that, while they and other staff members were often aware of parents' difficult emotions, there was no systematic process for acknowledging and assisting parents to deal with them.

Insinuations about intentional injury were seen to put additional stresses particularly on Māori and Pacific families. In the context of intensified media coverage of intentional injury amongst Māori at the time, a few reported that it was more likely that reference would be made to the hospital based child protection services (*Whakaruruhau) *when injured Māori and Pacific children were admitted to hospital than was the case with Pākehā children.

'Lots of times, because they are Māori or Pacific Island [children], Whakaruruhau tends to be talked about more. Sometimes that's quite a common trend... I don't know what sort of thing it has on the outcome, except that, if they do think of the family, that puts the whānau through a lot of stress.' (Kaitiaki focus group participant)

Such insinuations, even when shown to be unfounded, were perceived to have a lingering impact on staff attitudes.

'As a staff member it [the suggestion of abuse] definitely does cloud your thinking. Even if someone says "No it's not, it's been cleared", you can't help but have that logged into your brain. Is it or isn't it, type thing'. (Hospital nurse)

### Issues for Māori and Pacific family support services

A key role of the Kaitiaki and Pacific Family Support teams was providing practical and emotional support to Māori and Pacific families and resolving misunderstandings between families and staff. They identified a number of important issues for families and assisted where they could; however, they felt constrained at times in their ability to resource families in need. They did not have a budget of their own to draw from and, instead, had to get approval from the social worker or, if after hours, the duty manager.

*'I don't believe [we have] the full resources that we need if we have a wh*ā*nau that is stuck. OK, they've come in on an ambulance and they really don't have a vehicle to get home, they don't have a phone to get someone. Lots of those things are issues for our families. No telephone, no car. We've still got to go and ask for a taxi voucher to get them home.' (Kaitiaki focus group participant)*

One of the difficulties they experienced was that families often confused them with social workers, about whom they were very wary, and therefore were reluctant to engage their assistance.

'[Families] often mistake us for social workers and are scared we'll take away their benefit or take away their kids.' (Kaitiaki focus group participant)

Another perceived reason for families being wary of accessing their service was concern about confidentiality. This was an important consideration for Pacific families. For example, Pacific key informants reported that some families were hesitant about using an official interpreter for fear that their confidentiality would be compromised.

### Information needs

Key informants were very aware of the importance of good information as a means to empower children and families in the recovery and rehabilitative process. However, some felt that there was often not enough time to ensure parents and children did in fact understand what was told to them. They reported that sometimes parents received conflicting information from different professionals and that this was confusing.

'We give them a booklet [but] we need to go over the booklet to be sure they've got it... Sometimes there's too much, you can get too much information, but having it written down [helps]... And I think we forget that we just have to make it as simple as possible. Not too much information.' (Play therapist)

Moreover, there was a perceived lack of comprehensive written information for parents to take home.

'[We] constantly talk about giving information to families but our written information is not so good...It is written by people who are maybe nurses and doctors, they are good at being nurses and doctors, but are not necessarily good educators. So those are real gaps.' (Play therapist)

Where children were from other cultures or there were language barriers the importance of ensuring information was appropriately offered and understood was considered paramount.

'The other thing we don't do well at the moment, [but] we are actually investigating getting it done, is translating [information] into different languages other than English. Even if it's translated into Samoan, Tongan and Cantonese, for example, then at least we are covering a big area.' (Occupational therapist)

### Staff continuity and coordination issues

Poor staff continuity and coordination was a common theme. Each phase of the injured child's care from the emergency department to community rehabilitation had a different set of staff, and different methods of administration and data collection. Apart from there being no continuity of personnel for these phases, hand-over from one to the other was often not seamless. Consequently, key informants felt that parents experienced frustration when they had to repeat the same information to numerous staff.

'They arrive, are asked by the ambulance [officer], asked by the triage nurse, they are asked by the nurse that looks after them in ED, by the house surgeon, by the registrar, by the consultant and the same thing happens when they get to the ward. Everybody writes the same thing in the notes and, having been a parent who's stayed in hospital, it does become a little tedious'. (Hospital nurse)

The high staff turnover, especially of medical and nursing staff, created several problems to do with continuity and coordination of care. Medical House Surgeons changed three monthly and registrars six monthly. New staff members were often inexperienced or unfamiliar with 'unwritten' procedures and consequently certain processes, such as referrals, follow-up and coordinating with other health professionals in the ward setting, were not carried out as required. These issues were exacerbated during the winter months when the wards were very busy and many permanent staff members were away sick.

Another aspect of staff coordination related to professional boundaries. Key informants were respectful of the roles played by other professional groups and saw their roles as complementary. There was, however, recognition that staff needed to be constantly vigilant to ensure these relationships remained productive and that the children's needs were paramount.

'There is a tension between guarding your own profession - and that's a noble and a useful and a good thing - and also focusing on the needs of the child and seeking the best way to do that. So at times there are tensions. 'Is this something that a nurse should do or a physio should do?' But I think there are ways to dialogue those issues through and you keep them on the table.' (Hospital nurse)

### Issues after discharge from hospital

Issues of poor continuity between the hospital and community settings were also identified. Key informants felt that initial post-discharge supports were scarce. The brevity of the hospital stay was considered a contributing factor here. Rapid discharge and busy staff meant that parents were at times poorly equipped practically and emotionally to deal with caring for the child at home. Some parents were perceived to be still shocked about their child having had an accident and dealing with a range of emotions, including guilt and fear, that affected their decision-making and coping ability once their child was home. A rapid discharge without clear, well planned follow up and support was seen to place added strain on these families.

*'Children used to come in with a broken arm and they would be in here for a week. So you would have time to assist the parents through the shock. The shock of one minute your child is playing in the street and everything's fine and the next minute your life is so fragile. And not only is it fragile, this could have been your child dying. I think a lot of children are going home with parents who are still in that traumatic time themselves... I think we're not assessing. We are not asking these questions: how is it going to be for you in terms of managing? We ask the physical [ones], like, can you manage this child getting to the toilet and da-da-da? But we don't do such a good emotional stocktake*.' (*Play therapist)*

In recognition that families were discharged rapidly and might find the first days very difficult, several informants suggested that a follow-up phone call by hospital staff after the first 24 hours in order to monitor how the parents were coping and to offer advice and support would be helpful.

Exacerbating the early discharge issues was the difficulty of maintaining a strong and effective link between hospital and community services. Rotating hospital staff and the transience or short funding terms for some community agencies meant that any coordination that was established was often difficult to maintain. The well-recognised problems of non-attendance or difficulties in attending GP or out patients' clinics (e.g., transport, parking and long waiting time) were mentioned as ongoing issues of concern.

## Discussion

To our knowledge, this is the first study to provide in-depth, descriptive detail about the experiences and needs of children hospitalised for injury and their families from the perspective of health and support service providers. Strengths of the study included the wide range of key informant providers who willingly participated and the qualitative approach which enabled them to speak freely about their experiences and views. However, the findings must be interpreted in light of several limitations and the scope of this study.

The research was undertaken in the context of two large urban children's hospitals. The information gleaned may not be applicable to all other hospitals, particularly those where resources are even less accessible. Smaller hospitals in provincial or rural settings may, however, have closer engagement with their communities which could overcome some of the challenges identified in this study. Despite some changes to New Zealand's health and hospital services since the collection of these data, feedback from health professionals at presentations of the study findings at several national meetings in 2008 and 2009 suggest that many issues identified by key informants have persisted and are not unique to the hospitals involved in this research, or indeed, trauma services alone. The findings of the present study as well as feedback at presentations since indicate a high level of awareness among service providers regarding the many shortcomings of a busy and overloaded health service and empathy and insight into the innumerable issues faced by children and their families. Most felt they and their health service colleagues were doing the best they could within the constraints of an inadequately resourced system, whose services were stretched, particularly in clinical settings where relatively short-stay admissions are common.

It is possible that given the specific research questions explored in this study, participants were prompted to reflect particularly on service gaps and limitations rather than the areas in which the needs of children and families were adequately met. However, there was considerable agreement between the service issues and concerns identified by the key informants in this study and those mentioned by the whānau/families in the parallel strand of research in the larger study [24]. Both participant groups identified as issues; problems around information and communication needs, difficulties managing the multiple stresses on families when children are injured and hospitalised, the stressful impact of cultural stereotyping and the need for more appropriate resourcing for Māori and Pacific families. This high level of accord adds weight to their significance.

The study findings have direct implications for services that provide care for injured children and their families during and after hospitalisation. In general, the hospital system does not seem to adequately meet the needs of children with mild injuries. Evidences suggest that an injury which may be 'minor' with respect to threat to life, may have a considerable impact on the family involved as well as wider society [27]. Depending on the circumstances surrounding the event, the emotional consequences experienced by these children and their families can be extensive. A high prevalence of post traumatic stress disorder symptoms has been found in children with mild to moderate injury up to 18 months after the event [28]. Other studies, while not exclusively focusing on children, have reported that a considerable proportion of patients with minor traffic injuries had a slow recovery and long-term adverse psychological and social consequences. Psycho-social outcomes were found at times to linger for much longer than physical outcomes and were poorly predicted by the severity of the physical injury [29-31]. Several key informants were aware of this issue and perceived it as a function of time and resource constraints, rather than assuming that these children did not require much attention.

Key informants' concerns about the inadequacies of the hospital system in allaying parental distress appear well founded as one study found that parental distress played a more important role in the persistence of post traumatic stress disorder symptoms in children than the extent of the injury or the course of hospitalisation [28]. A number of factors contribute to parental distress during and after their child's hospitalisation. Important amongst these is inadequate information about the child's condition, care requirements and prognosis. Consistent with concerns expressed by families participating in the complementary arm of this study [32], key informants highlighted several problematic areas relating to communication, such as parents having to repeat the child's history often, language barriers and the need for better written information. Not alluded to by key informants but considered important by whānau/families was the need for appropriate and effective oral communications. Effective communication through a range of means is the cornerstone of ensuring patient health literacy, particularly where cultural miscommunications can arise [33]. Several studies have found that linguistic and cultural barriers adversely influence family involvement in the care of their children while in hospital and contribute to healthcare disparities for many minority groups [34-37].

The key informant Māori and Pacific support workers in the present study were aware of the significant stresses experienced by families of Māori and Pacific children who are known to experience a disproportionately high rate of unintentional injury [16-18]. However, they were concerned about resource constraints that limited the assistance they could offer these families, and the reality that their service was not accessible to families of most children with 'minor' injuries admitted for short periods This indicates that while the existence of such support services is crucial, these must be well resourced and extend to effective service delivery that is supported by an organisational culture that is responsive to the needs of these populations.

Problems with the coordination of services both within the hospital setting and between the hospital and the community were noted. The movement of patients between services is typically a difficult area to streamline but, in the case of an injury, the involvement of a number of professionals coupled with rapid discharge appears to exacerbate this difficulty. The key informants recognised the first 24 hours at home following discharge as a crucial transition phase. During this period, the 'second crisis of injury' may occur when responsibility shifts from professional care givers to family members [38]. Many informants felt that there was more that could be done of a relatively minor nature that could reduce the stress of this transition for families. For example, a simple follow-up phone call to check on how the child was, how the family was coping and to answer any questions was a frequent recommendation. The communication and coordination between hospital and community or educational services were also recognised as areas that needed more attention. These concerns suggest the need for carefully developed discharge plans that provide continuity of care and effectively work across the boundaries between hospital, home and school, encouraging and reinforcing well developed relations between hospital and community based care services.

For all of the reasons noted above, it is critical that information about hospital processes, issues that would become problematic following discharge to the community, and the resources available to support children and their families to negotiate these transitions, is given in a systematic and proactive manner rather than on an ad hoc basis. This would require the use of a variety of mechanisms to ensure that families under stress understand and retain necessary information. These are likely to include the need for culturally appropriate communication both orally and using written material, informative discharge plans, and where relevant, referrals to primary care and social services. It would be particularly important to ensure that all injured children and families who require further follow up by case managers in New Zealand's Accident Compensation Corporation (ACC), have access to this service. While the resources of this government-funded insurance scheme can serve as a potentially useful safety net for all New Zealanders requiring post-injury health care and rehabilitation, inequities in access to services by some ethnic groups, particularly including Māori, are well documented [39].

The accord between issues identified by the key informants in this study and those mentioned by the whānau/families in the parallel strand of research [24] suggests that many staff working with injured children and their families have a keen awareness of what constitutes optimal care but because of structural, time and other constraints are not always able to deliver it. The tension between providers' perceptions of optimum care and their ability to provide it is clearly an area for ongoing exploration and development. Systemic factors can provide significant barriers to effective care, however, health service providers' attitudes and behaviours also remain important influences on families' experience of health services and on the quality of care received [33,40]. In our study staff members' judgements around the cause of injury were a case in point.

Barriers to quality care for children hospitalised for injury are complex and multi factorial and a clear understanding and analysis of these barriers is needed if service improvements are to occur. Systemic, health provider and patient factors have been identified as key barriers to quality services [41] and interventions at these three levels have recently been proposed as a means to reduce ethnic disparities in the quality of trauma care [42]. Strategies that would appear to have particular relevance for our suggested improvements to services for children hospitalised for injury and their families include cultural competency training with a focus on patient health literacy, and clinical audits with feedback loops that support continuous quality improvement. Research designed to quantify the importance and predisposing factors that underlie the issues identified in this study could provide useful guidance to prioritise areas of action.

## Conclusions

Key informants provided valuable insights into key issues and concerns for children hospitalised for unintentional injury and their families within their own service and within those services with which they interacted. Given the significant impact of unintentional injury on childhood mortality and morbidity, improvement in the quality of health care and follow-up services is required to meet the needs of injured children and their families. Such initiatives might include focusing more attention on children with minor injuries, dealing with stresses of injury hospitalisations on families, ensuring more effective and appropriate communication of information, ensuring provider cultural competency and more effective support services for Māori and Pacific whānau/families and other ethnic groups and improving coordination of services both within the hospital setting and between the hospital and the community. These recommendations for service quality improvements for injured children include interventions at systemic, provider and patient levels, recognising that barriers to service quality operate at these levels and that a multi pronged approach to address them is likely to be most effective.

## Competing interests

The authors declare that they have no competing interests.

## Authors' contributions

SAm, SAb and SC conceived of and designed the study and obtained funding support. SAm and SAb analysed the data and drafted the manuscript as joint lead authors. STT assisted with the data analysis and drafting of the manuscript. LA and SM participated in the conduct of the study and data collection. All authors critically reviewed the draft manuscript, and read and approved the final manuscript.

## Authors' Information

SAm is based at the Section of Epidemiology & Biostatistics, School of Population Health, University of Auckland. All other authors were based at the Department of Maori & Pacific Health of the Faculty of Medical & Health Sciences, University of Auckland, during the conduct of this study. LA is now based at the Centre for Social and Health Outcomes Research & Evaluation, Massey University.

## Pre-publication history

The pre-publication history for this paper can be accessed here:

http://www.biomedcentral.com/1472-6963/10/333/prepub
